# Acute Bilateral Femoral Arterial Thrombosis in a Young Adult: A Rare Association With Asymptomatic COVID-19

**DOI:** 10.7759/cureus.14674

**Published:** 2021-04-25

**Authors:** Yusuf Parvez, Farheen Khan

**Affiliations:** 1 Pediatric Medicine, Dubai Hospital, Dubai, ARE

**Keywords:** covid-19, thrombotic, hypercoagulability, femoral artery

## Abstract

Thrombotic complications have been well described in patients with coronavirus disease 2019 (COVID-19) because of its associated states of hypercoagulability and inflammation. We present a unique case of thrombosis of the femoral artery in a 42-year-old man with asymptomatic COVID-19. This case highlights the need for vigilance when treating patients with COVID-19 to make the necessary diagnostic evaluations and provide appropriate treatment.

## Introduction

Coronavirus disease 2019 (COVID-19) has caused significant mortality and morbidity in various age groups. The disease has caused unexpected complications involving various organs of the human body. Although thromboembolism caused by COVID-19 has been well described in the literature, few cases of femoral arterial thrombosis have been reported [1]. We report a unique case of femoral arterial thrombosis to highlight the importance of awareness among healthcare providers when treating COVID-19 patients.

## Case presentation

**Figure 1:**
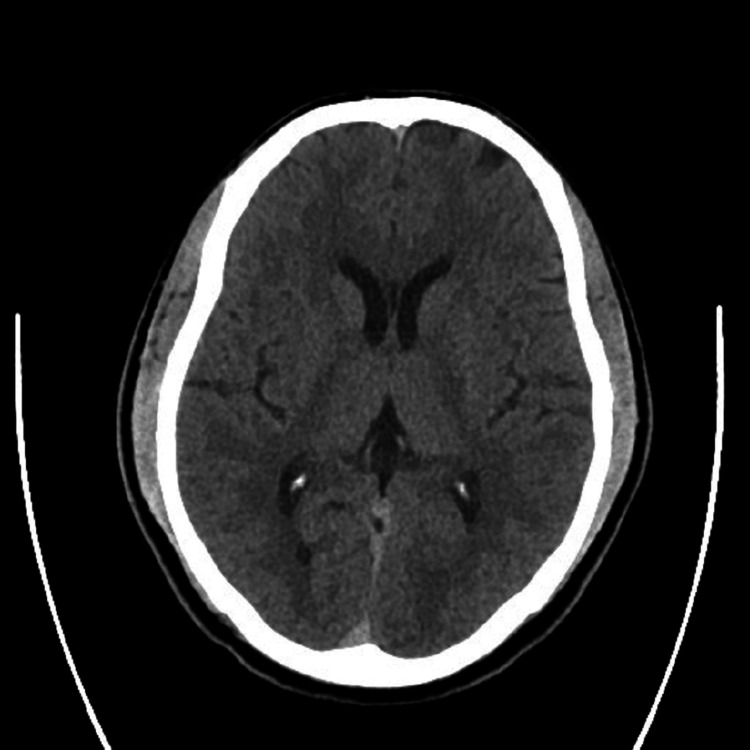
Computed tomography scan of the brain.

A 42-year-old previously healthy African man presented to our emergency department with sudden onset of pain in both legs and an inability to walk for one hour prior to presentation. He reported no history of cough, fever, difficulty breathing, or rashes. He had no previous history of joint pain or systemic illness. On presentation, he was conscious and oriented, both of his lower limbs were cold, and bilaterally, his femoral pulse was notable but not his distal pulse. Urgent computed tomography (CT) scan of the brain was unremarkable (Figure 1). His initial laboratory tests revealed D-dimer level of 14.03 µg/mL, ferritin of 1,399.7 ng/mL, creatinine phosphokinase of 5,736 U/L, creatinine of 2.3 mg/dL, and urea of 100 mg/dL. His polymerase chain reaction test result for COVID-19 was positive. Because his creatinine level was high, a nephrologist advised hydration. Because the high creatinine levels prohibited a CT angiogram, the vascular surgeon performed an urgent hand-held Doppler study, on which no signal was found. The patient was taken to the operation theater for an urgent embolectomy. He was also given 5,000 IU heparin prior to the embolectomy.

**Figure 2:**
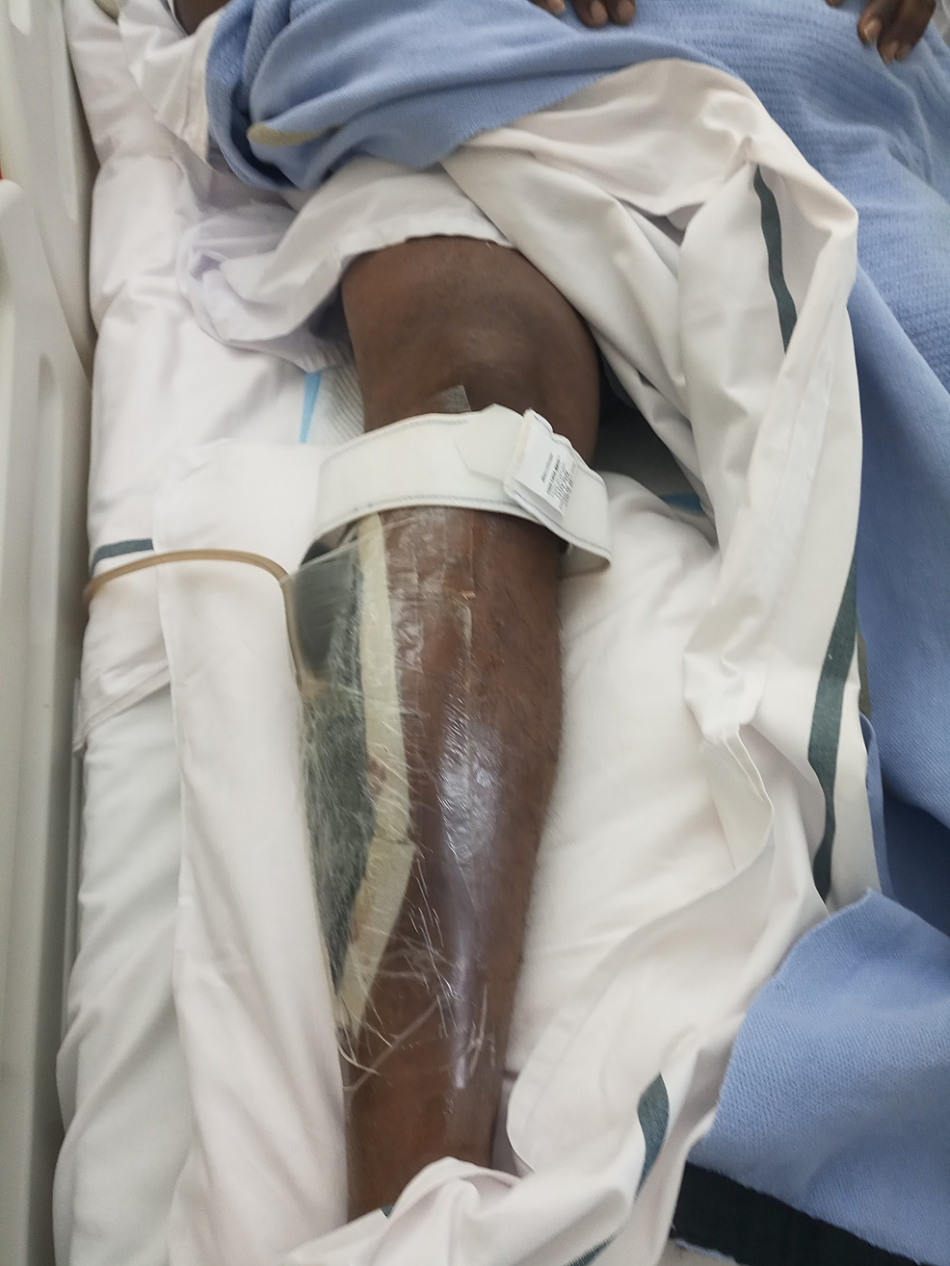
Lower limb with drain in situ postembolectomy.

A fresh embolus in both common femoral arteries and a fresh thrombus from the deep and superficial femoral arteries were removed during the surgical procedure. We administered 8,000 IU of heparin intraoperatively. Postoperatively, the patient had biphasic Doppler signals in the dorsalis pedis artery and the tibialis posterior artery in both legs, and monophasic signals in the anterior tibialis artery. He continued anticoagulant therapy, and a drain was inserted in both legs (Figure 2). He continued to recover and was discharged home after 10 days of hospitalization with advise for regular follow-up in the clinic.

## Discussion

Coronaviruses are a group of viruses that affect animals, birds, and humans [2]. Outbreaks of coronaviruses have been devastating, claiming many human lives. The severe acute respiratory syndrome coronavirus (SARS-CoV), the Middle Eastern respiratory coronavirus, and the recent SARS-CoV-2 (the virus responsible for COVID-19) have caused severe health concerns across the world [3].

COVID-19 mainly affects the respiratory system and causes life-threatening complications with significant mortality and morbidity. However, gastrointestinal, renal, cardiac, neurological, and ocular complications have also been described in the literature and should not be ignored. Involvement of the vascular system is thought to contribute significantly to morbidity and, more importantly, mortality. COVID-19-associated coaglulopathy, also known as thrombo-inflammation, has been well substantiated in the existing literature and explained by Virchow’s triad: endothelial injury, blood stasis, and hypercoagulability [4]. Various mechanisms have been described regarding the risk of thromboembolism in COVID-19. Elevated inflammatory markers (particularly interleukin-6), activation of angiotensin-2 receptors in endothelial cells, elevated factor VIII and D-dimer levels, and prolonged immobilization in an intensive care unit (ICU) are some of the contributing factors leading to thromboembolism in COVID-19 [5-7]. However, the acute complication of arterial thromboembolism of the femoral arteries due to a SARS-CoV-2 infection has rarely been described in the literature [1].

Treatment consists of anticoagulation and antiaggregant drugs and thrombectomy. Preventing venous thromboembolism is recommended in case of severe infection and for patients in the ICU; however, there is no clear recommendation for the prevention of arterial thromboembolism [8]. This case highlights the importance of an arterial event risk even if the infection is nonsevere and the patient is young.

## Conclusions

COVID-19 has caused significant mortality and morbidity in human beings involving multiple systems in the body. Thromboembolic disorders in asymptomatic SARS-CoV-2 infection are unusual presentations but should be kept in mind while dealing with COVID-19 patients. Appropriate investigations and treatments should be planned accordingly. Further prospective studies are warranted to substantiate the exact etiopathogenesis of thromboembolic disorders associated with COVID-19; nevertheless, thromboprophylaxis with low-molecular-weight heparin would be a prudent choice to prevent the complications.
